# Prospects for developing allergen‐depleted food crops

**DOI:** 10.1002/tpg2.20375

**Published:** 2023-08-28

**Authors:** Vadthya Lokya, Sejal Parmar, Arun K. Pandey, Hari K. Sudini, Dongxin Huai, Peggy Ozias‐Akins, Christine H. Foyer, Chogozie Victor Nwosu, Barbara Karpinska, Alison Baker, Pei Xu, Boshou Liao, Reyazul Rouf Mir, Xiaoping Chen, Baozhu Guo, Henry T. Nguyen, Rakesh Kumar, Sandeep K. Bera, Prashant Singam, Anirudh Kumar, Rajeev K. Varshney, Manish K. Pandey

**Affiliations:** ^1^ International Crops Research Institute for the Semi‐Arid Tropics (ICRISAT) Hyderabad India; ^2^ College of Life Science of China Jiliang University (CJLU) Hangzhou China; ^3^ Key Laboratory of Biology and Genetic Improvement of Oil Crops, Ministry of Agriculture and Rural Affairs Oil Crops Research Institute of the Chinese Academy of Agricultural Sciences Wuhan China; ^4^ Horticulture Department The University of Georgia Tifton Campus Tifton GA USA; ^5^ School of Biosciences College of Life and Environmental Sciences University of Birmingham Edgbaston UK; ^6^ MARS‐Wrigley Chicago IL USA; ^7^ Centre for Plant Sciences and School of Molecular and Cellular Biology, Faculty of Biological Sciences University of Leeds Leeds UK; ^8^ Division of Genetics and Plant Breeding Faculty of Agriculture Sher‐e‐Kashmir University of Agricultural Sciences and Technology Srinagar India; ^9^ Guangdong Provincial Key Laboratory for Crops Genetic Improvement Crops Research Institute of Guangdong Academy of Agricultural Sciences Guangzhou China; ^10^ USDA‐ARS, Crop Genetics and Breeding Research Unit Tifton GA USA; ^11^ Division of Plant Sciences and National Center for Soybean Biotechnology University of Missouri Columbia MO USA; ^12^ Department of Life Sciences Central University of Karnataka Gulbarga India; ^13^ ICAR‐Directorate of Groundnut Research Junagadh India; ^14^ Department of Genetics Osmania University Hyderabad India; ^15^ Central Tribal University of Andhra Pradesh Vizianagaram Andhra Pradesh India; ^16^ State Agricultural Biotechnology Centre, Crop Research Innovation Centre, Food Futures Institute Murdoch University Murdoch Western Australia Australia

## Abstract

In addition to the challenge of meeting global demand for food production, there are increasing concerns about food safety and the need to protect consumer health from the negative effects of foodborne allergies. Certain bio‐molecules (usually proteins) present in food can act as allergens that trigger unusual immunological reactions, with potentially life‐threatening consequences. The relentless working lifestyles of the modern era often incorporate poor eating habits that include readymade prepackaged and processed foods, which contain additives such as peanuts, tree nuts, wheat, and soy‐based products, rather than traditional home cooking. Of the predominant allergenic foods (soybean, wheat, fish, peanut, shellfish, tree nuts, eggs, and milk), peanuts (*Arachis hypogaea*) are the best characterized source of allergens, followed by tree nuts (*Juglans regia*, *Prunus amygdalus*, *Corylus avellana*, *Carya illinoinensis*, *Anacardium occidentale*, *Pistacia vera*, *Bertholletia excels*), wheat (*Triticum aestivum*), soybeans (*Glycine max*), and kidney beans (*Phaseolus vulgaris*). The prevalence of food allergies has risen significantly in recent years including chance of accidental exposure to such foods. In contrast, the standards of detection, diagnosis, and cure have not kept pace and unfortunately are often suboptimal. In this review, we mainly focus on the prevalence of allergies associated with peanut, tree nuts, wheat, soybean, and kidney bean, highlighting their physiological properties and functions as well as considering research directions for tailoring allergen gene expression. In particular, we discuss how recent advances in molecular breeding, genetic engineering, and genome editing can be used to develop potential low allergen food crops that protect consumer health.

## Core Ideas

- The severity and prevalence of food allergens varies between different geographical regions.
- Scope for developing hypoallergenic crops with minimal effects on plant physiology.
- Identification of crops with reduced allergen content through selection and breeding.

**Abbreviations**
AI: artificial intelligence
ATIs: α‐amylase/trypsin inhibitors
BAT: basophile activation tests
CAGR: compound annual growth rate
CRISPR: clustered regularly interspaced short palindromic repeats
FA: food allergy
Ig: immunoglobulin
NIAID: national institute of allergy and infectious diseases
nsLTPs: nonspecific lipid transfer proteins
PR: pathogenesis‐related proteins
RNAi: RNA interference
SDN: site‐directed nuclease
WHO/IUIS: World Health Organization and International Union of Immunological Societies

## 1 INTRODUCTION

Food allergy (FA) is a type of food sensitivity that has become a critical public health concern globally (Loh & Tang, 2018). FA describes the adverse immune responses to certain foods that can occur in the human body (Burks et al., 2012). The prevalence of FA has increased in recent decades, challenging both allergists and food scientists to devise rapid and accurate diagnostic tests, as well as prevention and treatment measures for vulnerable people. FA has become a global food safety issue because of the lack of reliable preventive measures, except for desensitization by immunotherapy (Du Toit et al., 2015) and the use of adrenaline injections for anaphylactic reactions. FA negatively impacts the life of sensitive individuals because of the absence of effective allergen elimination methods.

**FIGURE 1:**
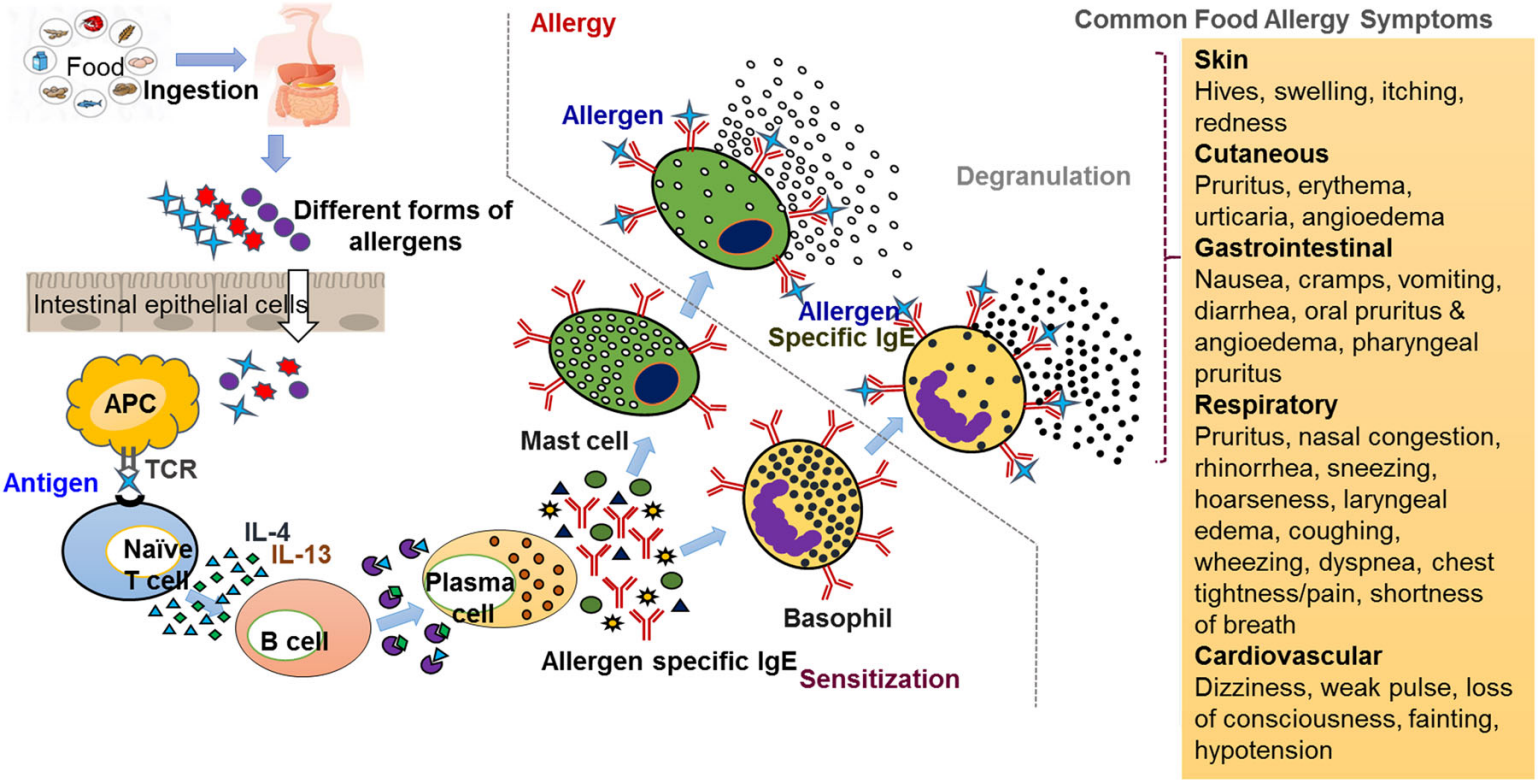
A pictorial representation of series of reactions involved in allergic immune response and most common allergic symptoms.

The generally accepted definition of the term “FA” is an “adverse health effect arising from a specific immune response that occurs reproducibly on exposure to a given food,” as described by the National Institute of Allergy and Infectious Diseases (NIAID) guidelines (Panel, 2010). In general, allergic disease manifestations are initiated through sensitization in early life and thereafter often progress into atopic dermatitis, asthma, allergic rhinitis, and other symptoms of FA. When a susceptible individual is exposed to food allergens for the first time, the offending food protein is identified by the body as a “foreign,” antigen. This results in the production of immunoglobulin E (IgE) antibodies, resulting in a “sensitization,” a state which results in no allergic symptoms. However, upon subsequent exposure, the specific allergens interact with the IgE molecules on the surface of tissue mast cells and basophils in the blood and trigger the release of a range of defense compounds, including histamine, prostaglandins, and leukotrienes, which together produce allergy symptoms (Figure 1; Sicherer & Sampson, 2014).

Food allergens are naturally occurring protein molecules that possess specific immunological characteristics and trigger inappropriate immune responses in susceptible individuals. In contrast, exposure of nonallergenic individuals to such food proteins in the gastrointestinal tract results in “oral tolerance” by producing protein‐specific IgG/IgA antibodies that develop immune response (Du Toit et al., 2015). Oral tolerance is a process of active suppression of the inappropriate immune responses to the first encounter with food protein antigens in the gastrointestinal tract (Tordesillas & Berin, 2018). Oral tolerance is considered to be the general homeostatic state, while FA is the exception. The balance between oral tolerance and FA can be influenced by several factors, such as genetic disposition, the dose, route and time of antigen exposure, dietary factors, and gastrointestinal microbiota (Keet & Wood, 2011). However, the complex, precise mechanism of oral tolerance and allergic sensitization to food proteins remains to be understood.

**FIGURE 2:**
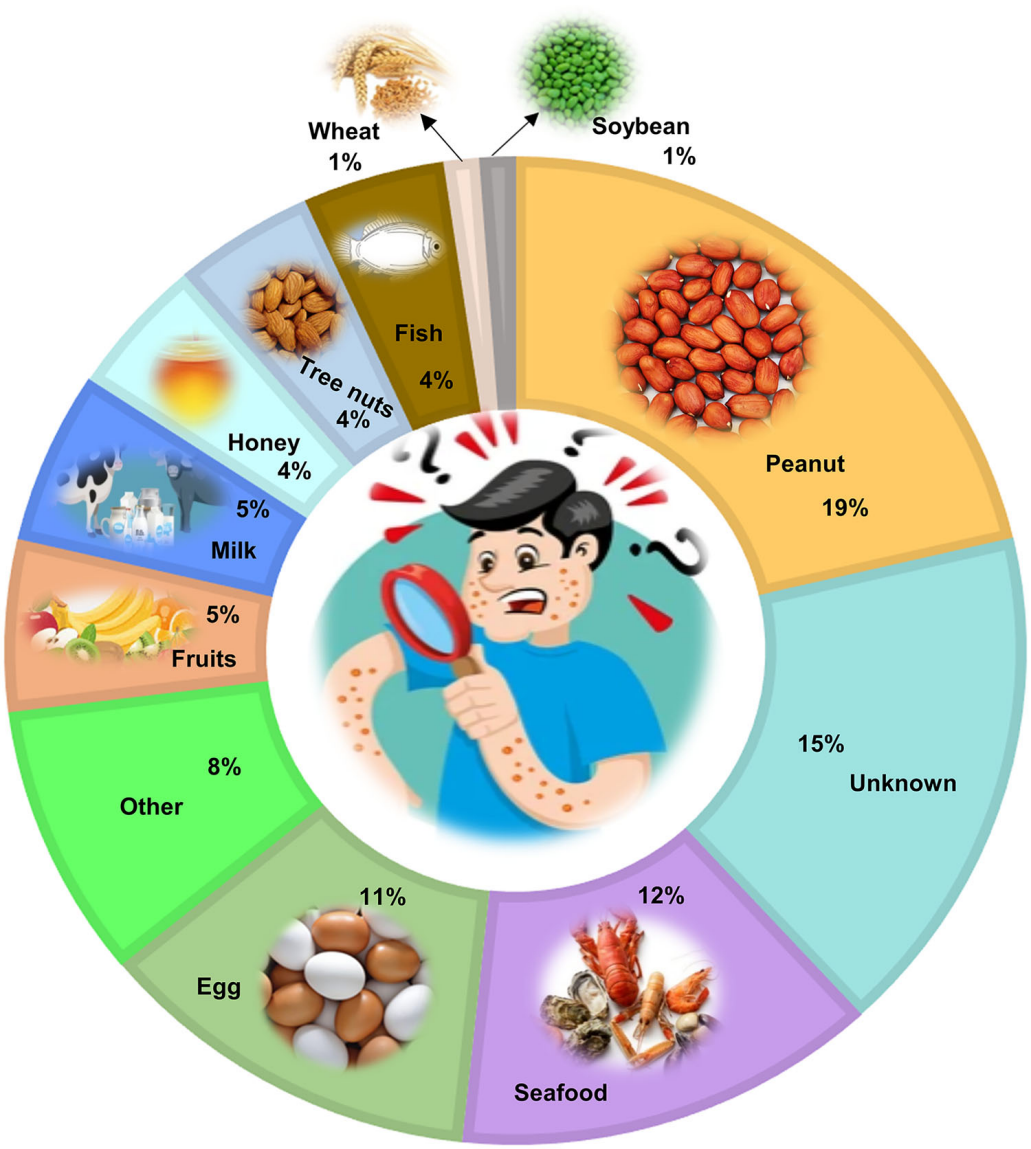
A pictorial representation of allergy composition associated with different foods of plant and animal origin and other unidentified sources.

The most common FA sources are of plant and animal origin, such as peanuts, tree nuts, fruits, wheat, soybean, kidney beans, seafood, eggs, milk, fish, honey, and other unidentified sources (Figure 2). Among different allergenic foods, peanuts cause the most common food allergies associated with severe anaphylaxis in children and adults and persist lifelong, which is uncommon in the case of other food allergies (Lieberman et al., 2020; Sicherer & Sampson, 2010). The allergic reaction can be unpredictable, such that the severity of health conditions ranges from mild to life threatening, the latter requiring immediate medical attention. Based on the immunological responses involved, FAs are classified as (i) IgE‐mediated, also called immediate hypersensitivity, (ii) non‐IgE‐ or cell‐mediated, also called delayed hypersensitivity, and (iii) a combination of IgE‐ and non‐IgE‐mediated allergic reactions (Tordesillas et al., 2017). Of these, IgE‐mediated FA is the most common type of allergy that is caused by the consumption of foods such as peanuts, tree nuts, wheat, shellfish, fish, eggs, milk, and soybeans and that can cause potentially fatal anaphylaxis (Wasserman et al., 2018). This involves an immediate Type I hypersensitivity reaction that develops within minutes to >2 h of ingestion of the offending food. The clinical manifestation of anaphylaxis involves multiple organs, such as the skin, gastrointestinal tract, respiratory tract and cardiovascular system. The most common symptoms associated with anaphylactic shock are abdominal cramps, nausea, vomiting, diarrhea, hives, itching, eczema, wheezing, and coughing, which can induce death due to respiratory and cardiovascular complications (Wasserman et al., 2018). In contrast, non‐IgE‐mediated FA does not involve IgE antibodies. In this case, symptoms often develop after 48–72 h of ingestion of offending foods such as cow's milk, soy, and wheat proteins. Non‐IgE‐mediated FA is usually resolved within 1–5 years. However, the mechanisms underlying non‐IgE‐mediated food allergic reactions are poorly understood perhaps because it is less harmful than IgE‐mediated anaphylaxis (Cianferoni, 2020).

Large numbers of allergenic food proteins have been identified and characterized, with specific details maintained in databases that are designed to provide easy access to comprehensive information. The “World Health Organization and International Union of Immunological Societies (WHO/IUIS) Allergen Nomenclature Database” was established at the millennium. The database contains a systematic nomenclature of allergenic proteins, as reviewed by the experts of the WHO/IUIS Allergen Nomenclature Sub Committee. Each entry in this database provides details on amino acid sequences, biochemical properties, and allergenicity. This reference database provides a crucial knowledge repository for the research community, clinicians, and regulatory authorities (Pomes et al., 2018). To date, on August 11, 2023, a total of 1015 (http://bioinfo.unipune.ac.in/AllerBase/PHP_codes/BrPlant.php) allergens have been experimentally validated from different plant sources and 1042 allergens from animal sources. This information is curated in the allergen database “AllerBase” (bioinfo.unipune.ac.in/AllerBase/Home.html), which provides data on allergens and their associated basic features, such as IgE binding epitopes, IgE antibodies, and cross‐reactivity. Inaddition, each allergen is curated with a cross‐reference to sequence and structure databases along with other allergen databases, such as the WHO/IUIS‐Allergen nomenclature database (www.allergen.org), Allergome (www.allergome.org), and AllFam (www.meduniwien.ac.at/allfam/) (Kadam et al., 2017). Understanding the correlation between allergen components in the food source and clinical symptoms has gained importance in the development of more accurate diagnostic methods which further help in the management of food allergies (Maruyama, 2021). Therefore, herein we have comprehensively described the prevalence of allergies associated with major plant food sources such as peanut (*Arachis hypogaea*), tree nuts (*Juglans regia*, *Prunus amygdalus*, *Corylus avellana*, *Carya illinoinensis*, *Anacardium occidentale*, *Pistacia vera*, *Bertholletia excels*), wheat (*Triticum aestivum*), soybean (*Glycine max*), and kidney bean (*Phaseolus vulgaris*) and also highlighted their physicochemical properties, functions as well as proposed future research directions for developing low allergenic plant food.

**Core Ideas**

- The severity and prevalence of food allergens varies between different geographical regions.
- Scope for developing hypoallergenic crops with minimal effects on plant physiology.
- Identification of crops with reduced allergen content through selection and breeding.

## 2 GLOBAL DISTRIBUTION OF FOOD ALLERGIES

**FIGURE 3:**
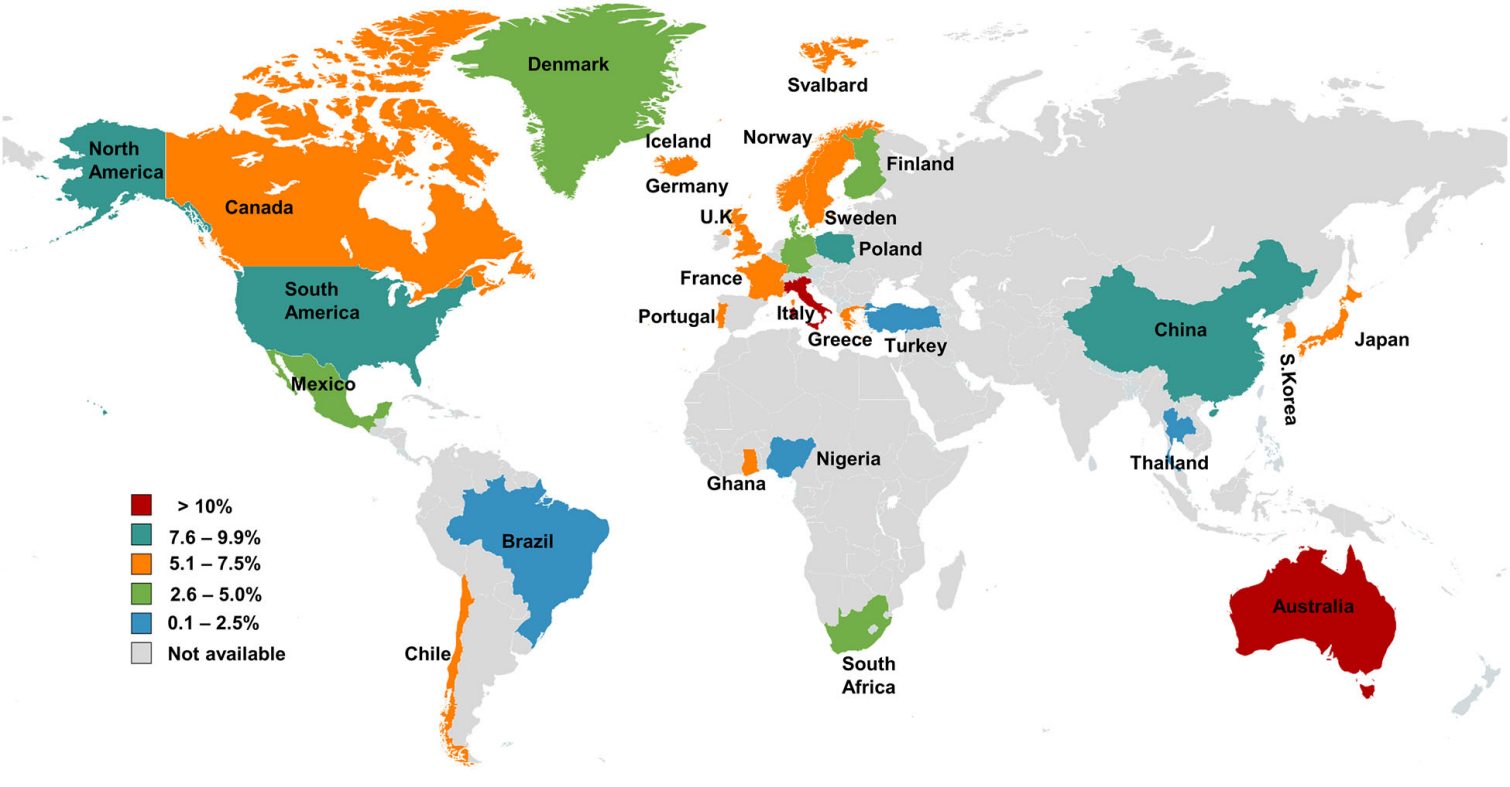
Global distribution of pediatric food allergy mostly children under the age of 5 years. *Source*: Center for Food & Asthma Research (CFAAR), Northwestern Feinberg School of Medicine, Ann & Robert H. Lurie Children Hospital of Chicago. A global map was obtained and modified to represent the current scenario of pediatric allergy.

The severity and prevalence of FAs vary between different geographical regions. According to WHO, the prevalence of FA has increased over the last two decades in industrialized countries, with similar trends observed in developing countries as their economic growth increases (Leung et al., 2018; Prescott et al., 2013). FA currently affects millions of people leading to restrictions in diet and daily activities, with profound emotional impacts, as well as healthcare and economic costs, lowering the overall quality of life as well as physical health and well‐being (Lieberman et al., 2020). Although the exact prevalence of FA is uncertain, it is estimated that over 220 million people suffered from some sort of FA globally (Sicherer & Sampson, 2018). The general consensus is that up to 10% people are affected by food allergies and that they are more common in young children than in adults (Loh & Tang, 2018). According to FAIR Health estimates, 5.9 million American children under the age of 18 have FA, which accounts for one in every 13 children or two in every classroom. In the case of Australia, the pediatric allergy prevalence ranges from 3.8 to 11% in children under the age of 4 years (Peters et al., 2017). Approximately 10% of Chinese children under the age of 6 years are also prone to FA (http://www.bjreview.com.cn/nation/txt/2015‐06/01/content_690263.htm). Similarly, the European Academy of Allergy and Clinical Immunology estimated that the allergy prevalence in European children is between 1.7 and 5%, depending on the country. It is matter of great concern that the majority of children in Australia, Italy, America, China, and Europe are predisposed to FA, followed by other nations, as illustrated in the global map (Figure 3). The incidence of peanut allergy is more prevalent in children in the United Kingdom, North America, and Australia. In these countries, the reported prevalence of peanut allergy has doubled over the last decade. The prevalence of FA is also increasing rapidly in Asian countries, correlating with increasing economic growth and westernization (Arakali et al., 2017). It is interesting to note that the prevalence of peanut allergies is lower in Asian countries; but have a prevalence of allergies to fish. The developing nations in Asia and Africa have gradually adopted a Western lifestyle and have observed increasing rates of allergic disease across age groups, particularly in younger children.

Obtaining accurate FA incidence data remains a major challenge in many parts of the world. Problems arise because of difficulties in data curation or a lack of consistency in study design, approach, specific population analysis, specific foods, and even the definition of FA, factors that together lead to a poor establishment of accurate prevalence statistics (Prescott et al., 2013). Currently, available statistical data are highly biased not least because they rely heavily on self‐reporting of FA incidence rather than challenge‐confirmed FA analysis. Such factors may result in over estimation of the allergy prevalence, often because of patient's misunderstanding about wide range of symptoms that are associated with particular food allergies (Woods et al., 2002). Researchers in westernized countries have tested numerous methods to determine the precise number of people that are allergic to specific foods (Prescott et al., 2013). Hospital intake data that include healthcare burden, insurance claims, and telephone surveys is the most frequently used method of evaluation of prevalence rates (Gupta et al., 2018a). However, comprehensive studies are needed in the future to demonstrate a precise correlation between different geographic regions and specific allergy prevalence. Moreover, the instigation of common dedicated databases at the clinic/hospital level is vital for recording the incidence of food allergies.

### 2.1 Specific regions and populations affected

There is a paucity of reliable data concerning FA prevalence, even in developed countries, and so it is difficult to compare prevalence rates between countries (Prescott et al., 2013). Significant variations in prevalence and causative foods have been reported. For example, in continental Europe, adults are predominantly allergic to peanuts, tree nuts, fruits, and vegetables but children are allergic to cow's milk, peanuts, and eggs (Baker, 2018). Moreover, kidney beans are the major allergic triggers of asthma and rhinitis patients in India, followed by chickpeas and peanuts (Kasera et al., 2011), whereas Scandinavians have an allergic sensitivity to shellfish and cod.

The overall prevalence of self‐reported FA in Europe is 6%, the most common causative foods being cow's milk, eggs, peanuts, tree nuts, wheat, soy, fish, and shellfish (Lyons et al., 2019). A systematic review of studies made between 2000 and 2012 revealed that the self‐reported lifetime FA prevalence of different plant food sources, such as wheat, tree nuts, peanut, and soy, was 3.6, 2.2, 1.3, and 0.4%, respectively. In contrast, the prevalence of food‐challenge‐defined allergies to wheat, tree nuts, peanut, and soy was 0.1, 0.5, 0.2, and 0.3%, respectively (Nwaru et al., 2014). It has been estimated that >17 million people are currently affected by FA, of which 3.5 million are under 25 years of age (Baker, 2018). The average healthcare cost of an allergic adult is estimated to be I$2016, compared with the cost of a healthy adult, which is I$1089 per year (Fox et al., 2013).

**FIGURE 4:**
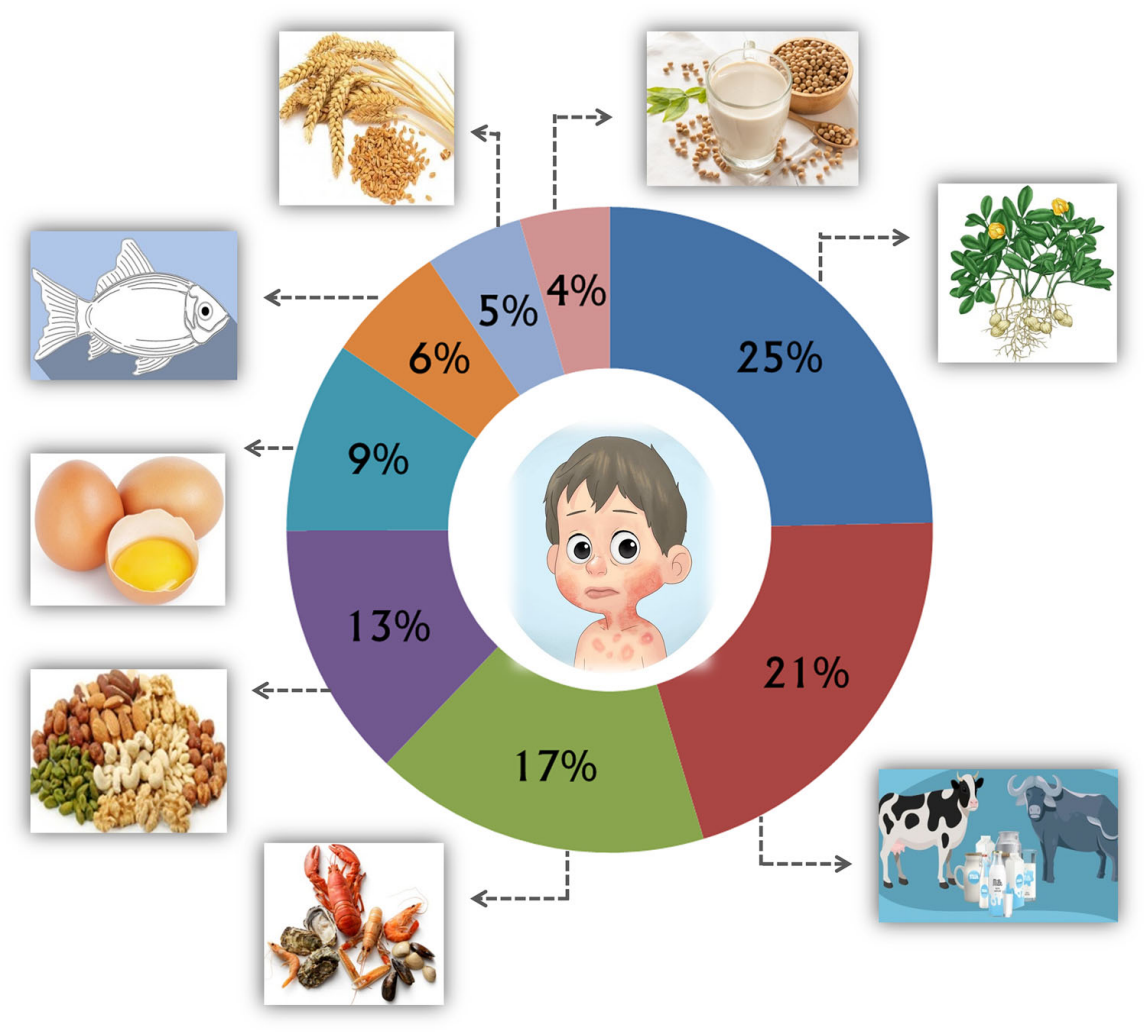
Prevalence of specific food allergy in allergic children of United States. Among the different food sources, peanut, milk, shellfish and tree nuts hold a major stake in contributing toward triggering life‐threatening hypersensitivity reactions in children.

Approximately 10% of the US population suffers from at least one form of IgE‐mediated FA, which affects about one in 10 adults and one in 13 children (Gupta et al., 2018a, 2019; Warren et al., 2020). The most common allergic foods include peanuts and tree nuts, which are associated with severe allergic reactions in all age groups. According to the FAIR health insurance claims‐based study, the incidence of severe anaphylactic reactions increased by 377% between 2007 and 2016 due to the consumption of peanuts, tree nuts/seeds, and other specific foods (Motosue et al., 2018). Peanut is a major contributor to life‐threatening anaphylaxis (26%), followed by tree nuts/seeds (18%), eggs (7%), crustaceans (6%), milk products (5%), fruits and vegetables (2%), and other specific foods. A population‐based survey involving 40,443 US adults (Gupta et al., 2019) suggested that 10.8% (equivalent to >26 million people) have some form of FA, while 19% of adults believed themselves to have food allergies. Shellfish allergies are particularly predominant in US adults, accounting for 7.2 million people (2.9%), followed by milk (4.7 million = 1.9%), peanut (4.5 million = 1.8%), tree nuts (3.0 million = 1.2%), finfish (2.2 million = 0.9%), egg (2.0 million = 0.8%), wheat (2.0 million = 0.8%), soy (1.5 million = 0.6%), and sesame (0.5 million = 0.2%)‐associated allergies. However, children appear to be more sensitive to peanuts, followed by milk, shellfish, tree nuts, eggs, finfish, wheat, soy, and sesame seeds (Gupta et al., 2011). The big eight foods and their specific allergy prevalence in children are as follows: peanut (25.2%), milk (21.1%), shellfish (17.2%), tree nuts (13.1%), egg (9.8%), finfish (6.2%), wheat (5%), and soy (4.6%) (Figure 4). The prevalence of FA in children aged 0–17 years increased by 50% from 1997 to 2011. This trend is similar to the increasing trend in increased income levels (Jackson et al., 2013). The overall economic cost of FA was estimated with be US$24.8 billion annually, which is equivalent to $4184 per child (Gupta et al., 2013).

The European Academy of Allergy and Clinical Immunology estimated the allergy prevalence in children to be 5% in France, the United Kingdom, Germany, and the Netherlands, 4% in Italy and Spain and 1.7% in Greece. In the case of the UK, 2 million people (1–2% of adults) and 5–8% of children exhibit food allergies. In Europe, there are 14 foods, which include celery, cereals, crustaceans, eggs, fish, lupin, milk, mollusks, mustard, peanuts, sesame, soybean, sulfur dioxide, and sulfites, which have been identified as major potent and prevalent allergens. In Australia, approximately 2% of adults and 4–8% of children have food allergies and there has been a fourfold increase in anaphylaxis hospitalizations (Tang & Mullins, 2017). The Health Nuts population‐based cohort study reported the highest prevalence of challenge‐confirmed FA in Australia. Overall 11% of Australian children at the age of 1 year and 3.8% at the age of 4 years had FA. Peanut was the predominant allergen at 1.9%, followed by egg (1.2%) and sesame (0.6%) in children at 4 years of age (Peters et al., 2017). Peanut allergy, which affects about 2% of the population of Western nations, has become a burden of self‐management to protect against accidental exposure.

The overall prevalence of FA in Asia follows similar trends but there are significant differences in the types of reported FA. Shellfish allergy is more prevalent than peanut allergy in Asia (Lee et al., 2013). There is an increasing prevalence of FA reported in children in developing parts of Asia, including Australia, Japan, China, and Korea. Between 1 and 2% of adults and 5% of children report FA in China (Poulos et al., 2007; Prescott et al., 2013). The Population Reference Bureau, China reported that 3.8–7.7% of children have food intolerances (Hu et al., 2020). This report identified shellfish, peanuts, soybeans, wheat, tree nuts, fish, eggs, and milk as a major source of allergens. Many prepacked foods contain these constituents and they are sold with proper labeling and instructions (Baker, 2018). Most allergic diseases in India are caused by pollen grains, fungal spores, insects, and foods. About 25% of the Indian population shows sensitivities to these different forms of allergens (Bhattacharya et al., 2018). The EuroParvall‐INCO survey conducted by Li et al. (2020) revealed that IgE‐mediated FA prevalence in children is highest in Hong Kong (1.5%), followed by Russia (0.87%), Guangzhou (0.21%) and Shaoguan (0.69%) of China, and India (0.14%). A similar study conducted on adults in Karnataka, India, revealed a high rate (26.5%) of sensitization but a low rate (1.2%) of probable FA (Mahesh et al., 2016). These studies suggest there is little correlation between food‐specific IgE sensitization and probable food allergies. This finding highlights the role of other important factors involved in the clinical manifestation of allergies.

The prevalence of food allergies varies between different ethnic groups. For example, children in Ghana (5–16 years of age) are commonly allergic to pineapple, pawpaw, orange, mango, and peanut (Obeng et al., 2011), while children in North America are allergic to peanut, milk, egg, shellfish, and soybean (Hill et al., 2016). Similarly, African‐American children have a higher prevalence than their Caucasian counterparts (Gupta et al., 2018a). While Asian‐American children have the lowest reported FA, Caucasians have the highest rates of diagnosed FA (Gupta et al., 2011). Additionally, the prevalence of FA varies between races, as well as rural and urbanized populations. For example, white adults in the US report lower rates of FA than their Asian, Hispanic, Black, and Multiracial counterparts (Gupta et al., 2019). Crucially, however, the emergency admission rates of food‐induced anaphylaxis were approximately three times higher in urban settings than in rural settings in New York and Florida (Gupta et al., 2012).

### 2.2 Sources of food allergens

**FIGURE 5:**
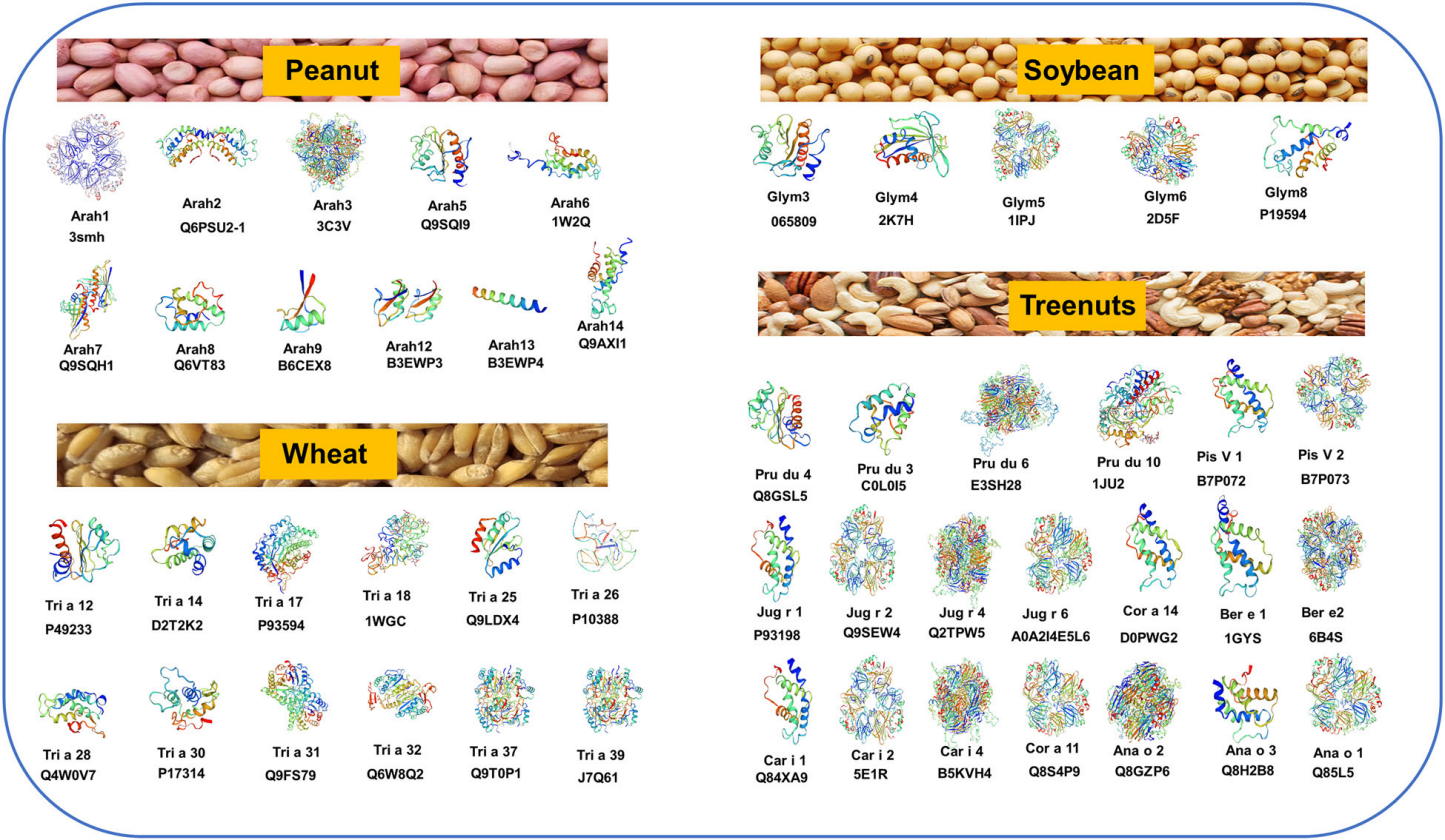
Different allergen protein structure of peanut, wheat, soybean, and treenuts. (*Source*: https://fermi.utmb.edu/cgi‐bin/SDAP/sdap_01).

Plants are the dominant source of cause of FA in most countries (Lyons et al., 2019). Plant allergenic proteins carry IgE binding epitopes that are partially or fully resistant to digestive proteolysis. These proteins act as antigens that trigger an unusual immune response. Peanuts are the most common source of life‐threatening allergens. Peanuts, tree nuts, wheat, and soybean are considered to contain a large number of allergenic proteins (Table 1 and Figure 5). Major tree nuts, including almond, walnut, cashew, hazelnut, pecan, pistachio, and brazilnut, are potent sources of IgE‐induced allergens and account for a prevalence of 4.9% worldwide (McWilliam et al., 2015). The tree nut allergens are primarily characterized by relatively few protein families, particularly 2S albumin, legumins, vicilins, nonspecific lipid transfer proteins (nsLTP), and Bet v 1‐homologs/profilins, which are associated with pollen tree nut allergies (Geiselhart et al., 2018). To date, 963 allergens have been isolated and characterized from different plant sources. Details concerning these allergens are maintained in the dedicated allergen database “AllerBase” (http://bioinfo.unipune.ac.in/AllerBase/Home.html).

**TABLE 1:** Protein families of different types of allergens from peanut, soybean, wheat, and major six tree nuts (walnut, almond, hazelnut, pecan, cashew, pistachio, Brazil nut).

Allergen protein	Isoallergen/variants	MW (kDa)	Uniprot Id	Allergen family	Biological function	References
*Arachis hypogaea* (17)						
Ara h 1	Ara h 1.0101	64	P43238	Cupin (Vicilin‐type, 7S globulin)	Seed storage protein and trypsin inhibitor activity might play role in plant defense against insect	Burks et al. (1995)
	Ara h 3.0101	61.0	O82580	Cupin (Legumin‐type, 11S globulin)	Seed storage protein and provide a store of amino acids for use during germination and seed growth	Rabjohn et al. (1999)
	Ara h 3.0201	57.0	Q9SQH7			Kleber‐Janke et al. (1999)
	Ara h 3	61.7	Q8LKN1			Viquez et al. (2004)
	iso‐Ara h 3	60.0	Q6IWG5			Boldt et al. (2005)
	Ara h 2.0101	16.7	Q6PSU2	Conglutin (2S albumin)	Seed storage protein and provide a store of amino acids for use during germination and seed growth	Stanley et al. (1997) and Viquez et al. (2001)
	Ara h 2.0201	18.1	Q6PSU2			Chatel et al. (2003)
	Ara h 6.0101	14.5	Q647G9			Kleber‐Janke et al. (1999)
	Ara h 7.0101	16.3	Q9SQH1			Kleber‐Janke et al. (1999)
	Ara h 7.0201	17.4	B4XID4			Schmidt et al. (2009)
	Ara h 7.0301	17.3	Q647G8			Yan et al. (2005)
	Ara h 5.0101	15.0	Q9SQI9	Profilin	Cytoskeletal and membrane trafficking	Kleber‐Janke et al. (1999) and Kleber‐Janke et al. (2001)
	Ara h 8.0101	16.9	Q6VT83	Pathogenesis related (PR‐10)	Unclear	Mittag et al. (2004)
	Ara h 8.0201	16.3	B0YIU5			Riecken et al. (2008)
	Ara h 9.0101	9.1	B6CEX8	Nonspecific lipid transfer protein (nsLTP)	Stabilization of membrane, cell wall organization, signal transduction, and plant development	Krause et al. (2010)
	Ara h 9.0201	9.0	B6CG41			Krause et al. (2010)
	Ara h 16	8.5	–			–
	Ara h 17	11.0	–			–
	Ara h 10.0101	16.0	Q647G5	Oleosin	Lipid and energy metabolism	Schwager et al. (2015)
	Ara h 10.0102	16.0	Q647G4			Schwager et al. (2015)
	Ara h 11.0101	14.0	Q45W87			Schwager et al. (2015)
	Ara h 11.0102	14.0	Q45W86			Schwager et al. (2015)
	Ara h 14.0101	18.4	Q9AXI1			Pons et al. (2005) and Schwager et al. (2015)
	Ara h 14.0102	18.4	Q9AXI0			Pons et al. (2005) and Schwager et al. (2015)
	Ara h 14.0103	18.4	Q6J1J8			Schwager et al. (2015)
	Ara h 15.0101	17.0	Q647G3			Schwager et al. (2015)
	Ara h 12.0101	5.2	B3EWP3		Plant defensing and antifungal activity and also showed inhibitory activity on mold strains	Petersen et al. (2015)
	Ara h 13.0101	5.5	EY396019	Defensins		Petersen et al. (2015)
	Ara h 13.0102	5.5	COHJZ1			Petersen et al. (2015)
	Ara h 18.0101	21	A0A444XS96	Cyclophilin‐peptidyl‐prolyl cis‐trans isomerase	Unclear	Allergen.org
*Glycine max* (8)						
Gly m 1	Gly m 1.0101 Gly m 1.0102	7	Q9S8F3 Q9S8F2		Hydrophobic protein	Gonzalez et al. (1991)
Gly m 2	Gly m 2.0101	8	–		Defensins	Codina et al. (1997)
Gly m 3	Gly m 3.0101 Gly m 3.0102	14	O65809 O65810		Profilin	Rihs et al. (1999)
Gly m 4	Gly m 4.0101	17	P26987		Pathogenesis‐related protein, PR‐10, Bet v 1 family member	Crowell et al. (1992)
Gly m 5	Gly m 5.0101 Gly m 5.0201 Gly m 5.0301 Gly m 5.0302	–	022120 Q9FZP9 P25974 P25974		Beta‐conglycinin (vicilin, 7S globulin)	AB_P_00904 (AllerBase ID)
Gly m 6	Gly m 6.0101 Gly m 6.0201 Gly m 6.0301 Gly m 6.0401 Gly m 6.0501	–	P04776 P04405 P11828 Q9SB11 Q7GC77		Glycinin (legumin, 11S globulin)	AB_P_00906 (AllerBase ID)
Gly m 7	Gly m 7.0101	76.2	C6K8D1		Seed biotinylated protein	Riascos et al. (2010)
Gly m 8	Gly m 8.0101	28	P19594		2S albumin	Ebisawa et al. (2013) Klemans et al. (2013)
*Triticum aestivum* (28)						
Tri a 12	Tri a 12.0101 Tri a 12.0102 Tri a 12.0103 Tri a 12.0104	14	P49232 P49233 P49234 B6EF35		Profilin, actin binding protein	AB_P_01828 (AllerBase ID)
Tri a 14	Tri a 14. 0101 Tri a 14. 0201	9	D2T2K2		Nonspecific lipid transfer protein 1	Sander et al. (2011)
Tri a 15	Tri a 15.0101		D2TGC3		Monomeric alpha‐amylase inhibitor	Sander et al. (2011)
Tri a 17	Tri a 17.0101	56	P93593		Beta‐amylase	AB_P_01881 (AllerBase ID)
Tri a 18	Tri a 18.0101	–	P10968		Agglutinin isolectin 1	Sutton et al. (1984)
Tri a 19	Tri a 19.0101	65	Q40215		Omega‐5 gliadin, seed storage protein	Lehto et al. (2003)
Tri a 20	Tri a 20.0101	35‐38	A0A060N479		Gamma gliadin	Yokooji et al. (2013)
Tri a 21	Tri a 21.0101	–	D2T2K3		Alpha‐beta‐gliadin	Sander et al. (2011)
Tri a 25	Tri a 25.0101	–	Q9LDX4		Thioredoxin	Weichel et al. (2006)
Tri a 26	Tri a 26.0101 Tri a 26.0201	88	P10388 Q45R38		High‐molecular‐weight glutenin	AB_P_01837 (AllerBase ID)
Tri a 27	Tri a 27.0101	27	Q7Y1Z2		Thiol reductase homoloque	Sander et al. (2011)
Tri a 28	Tri a 28.0101	13	Q4W0V7		Alpha‐amylase inhibitor	Sander et al. (2011)
Tri a 29	Tri a 29.0101 Tri a 29.0201	13	C7C4 × 0 D2TGC2		Alpha‐amylase inhibitor	Sander et al. (2011)
Tri a 30	Tri a 30.0101	16	P17314		Alpha‐amylase inhibitor	Sander et al. (2011)
Tri a 31	Tri a 31.0101	–	Q9FS79		Triosephosphate‐isomerase	Sander et al. (2011)
Tri a 32	Tri a 32.0101	–	Q6W8Q2		1‐Cys‐peroxiredoxin	Sander et al. (2011)
Tri a 33	Tri a 33.0101	–	Q9ST57		Serpin	Sander et al. (2011)
Tri a 34	Tri a 34.0101	–	C7C4 × 1		Glyceldehyde‐3‐phosphate dehydrogenase	Sander et al. (2011)
Tri a 35	Tri a 35.0101	–	D2TE72		Dehydrin	Sander et al. (2011)
Tri a 36	Tri a 36.0101	40	B2Y2Q7		Low‐molecular‐weight glutenin GluB3‐23	AB_P_01847 (AllerBase ID)
Tri a 37	Tri a 37.0101	12	Q9T0P1		Alapa purothionin	AB_P_01848 (AllerBase ID)
Tri a 39	Tri a 39.0101	–	J7QW61		Serine protease inhibitor	Sander et al. (2015)
Tri a 40	Tri a 40.0101	15.96	Q41540		Alpha‐amylase inhibitor	Sander et al. (2016)
Tri a 41	Tri a 41.0101	–	A0A0G3F2P1		Mitochondrial ubiquitin ligase activator of NFKB 1	AB_P_01852 (AllerBase ID)
Tri a 42	Tri a 42.0101	–	A0A0G3F2F5		Hypothetical protein	AB_P_01853 (AllerBase ID)
Tri a 43	Tri a 43.0101	–	A0A0G3F5F7		Hypothetical protein	AB_P_01854 (AllerBase ID)
Tri a 44	Tri a 44.0101	–	A0A0G3F720		Endosperm transfer cell specific PR60 precursor	AB_P_01855 (AllerBase ID)
Tri a 45	Tri a 45.0101	–	A0A0G3F715		Elongation factor 1	AB_P_01856 (AllerBase ID)
*Juglans regia* (8)						
Jug r 1	Jug r 1.0101	15‐16	P93198		2S albumin seed storage protein	Teuber et al. (1998)
Jug r 2	Jug r 1.0101 Jug r 2.0101	44	Q9SEW4 Q9SEW4		Vicilin seed storage protein	Teuber et al. (1998)
Jug r 3	Jug r 3.0101	9	C5H617		Nonspecific lipid transfer protein 1 (nsLTP1)	Pastorello et al. (2004)
Jug r 4	Jug r 4.0101	58.1	Q2TPW5		11S globulin seed storage protein	Wallowitz et al. (2006)
Jug r 5	Jug r 5.0101	20	AOA1JORET5		PR‐10	AB_P_01052 (AllerBase ID)
Jug r 6	Jug r 6.0101	47	AOA214E5L6		Vicilin‐like cupin	Dubiela et al. (2018)
Jug r 7	Jug r 7.0101	13	AOA214DNN6		Profilin	AB_P_01054 (AllerBase ID)
Jug r 8	Jug r 8.0101 Jug r 8.0101	9	AOA214EB91 AOA214GT96		nsLTP‐2	AB_P_02015 (AllerBase ID)
*Prunus amygdalus* (6)						
Pru du 3	Pru du 3.0101	9	C0L0I5		nsLTP1	AB_P_01557 (AllerBase ID)
Pru du 4	Pru du 4.0101 Pru du 4.0102	14	Q8GSL5 Q8GSL5		Profilin	Tawde et al. (2006)
Pru du 5	Pru du 5.0101	10	Q8H2B9		60s acidic ribosomal protein 2	AB_P_01559 (AllerBase ID)
Pru du 6	Pru du 6.0101 Pru du 6.0201	360	E3SH28 E3SH29		Amandin, 11S globulin legumin‐like protein	Kabasser et al. (2021)
Pru du 8	Pru du 8.0101	31	A0A516F3L2		Antimicrobial seed storage protein	Chw et al. (2019)
Pru du 10	Pru du 10.0101	60	Q945K2		Mandelonitrile lyase 2	Kabasser et al. (2021)
*Corylus avellana* (11)						
Cor a 1	Cor a 1.0101 Cor a 1.0102 Cor a 1.0103 Cor a 1.0104 Cor a 1.0201 Cor a 1.0301 Cor a 1.0401 Cor a 1.0402 Cor a 1.0403 Cor a 1.0404	17	Q08407 Q08407 Q08407 Q08407 Q39453 Q39454 Q9SWR4 Q9FPK4 Q9FPK3 Q9FPK2		PR‐10, Bet v 1 family member	Pastorello et al. (2002)
Cor a 2	Cor a 2.0101	14	Q9AXH5 Q9AXH4		Profilin	Lauer et al. (2004)
Cor a 6	Cor a 6.0101	35	A0A0U1VZC8		Isoflavone reductase	AB_P_00558 (AllerBase ID)
Cor a 8	Cor a 8.0101	9	Q9ATH2		nsLTP type 1	Pastorello et al. (2002)
Cor a 9	Cor a 9.0101	40	Q8W1C2		11S seed storage globulin	Beyer et al. (2002)
Cor a 10	Cor a 10.0101	70	Q9FSY7		Luminal binding protein	Gruehn et al. (2003)
Cor a 11	Cor a 11.0101	48	Q8S4P9		7S seed storage globulin	Lauer et al. (2004)
Cor a 12	Cor a 12.0101	17	Q84T21		Oleosin	AB_P_00554 (AllerBase ID)
Cor a 13	Cor a 13.0101	14‐16	Q84T91		Oleosin	AB_P_00555 (AllerBase ID)
Cor a 14	Cor a 14.0101	10	D0PWG2		2S albumin	Garino et al. (2010)
Cor a 15	Cor a 15.0101	17	–		Oleosin	AB_P_02079 (AllerBase ID)
*Carya illinoinensis* (3)						
Car i 1	Cari i 1.0101	16	Q84XA9		2S albumin seed storage protein	Sharma et al. (2011)
Car i 2	Cari i 2.0101	55	B3STU4		Vicilin‐like protein	Zhang et al. (2016)
Car i 4	Cari i 4.0101	55.4	B5KVH4		Legumin seed storage protein	Sharma et al. (2011)
*Anacardium occidentale* (3)						
Ana o 1	Ana o 1.0101 Ana o 1.0102	50	Q8L5L5 Q8L5L6		Vicilin‐like protein	Wang et al. (2002)
Ana o 2	Ana o 2.0101	55	Q8GZP6		Legumin‐like protein	Wang et al. (2003)
Ana o 3	Ana o 3.0101	14	Q8H2B8		2S albumin	Robotham et al. (2005)
*Pistacia vera* (5)						
Pis v 1	Pis v 1.0101	7	B7P072		2S albumin	Ahn et al. (2009)
Pis v 2	Pis v 2.0101 Pis v 2.0201	32	B7P073 B7P074		11S globulin	Ahn et al. (2009)
Pis v 3	Pis v 3.0101	55	B4 × 640		Vicilin	Willison et al. (2008)
Pis v 4	Pis v 4.0101	25.7	B2BDZ8		Manganese superoxide dismutase	Noorbakhsh et al. (2010)
Pis v 5	Pis v 5.0101	36	B7SLJ1		11S globulin	AB_P_01464 (AllerBase ID)
*Bertholletia excels* (2)						
Ber e 1	Ber e 1.0101	9	P04403		2S sulfur rich seed storage albumin	Alcocer et al. (2012)
Ber e 2	Ber e 2.0101	29	Q84ND2		11S globulin seed storage protein	AB_P_00284 (AllerBase ID)

Many food plants contain proteins that are referred to as lectins based on their specific carbohydrate‐binding properties. Lectins are major antinutritional factors in seeds. For example, the most abundant lectin family proteins are arcelin, phytohemagglutinin, and a‐amylase inhibitor–APA proteins) in common bean (*P. vulgaris*). The major antinutritional effects of these proteins are caused by their low digestibility and high toxicity in the intestinal tract (Bardocz et al., 1995). Four allergen proteins in kidney beans (alpha‐amylase inhibitor precursor, phaseolin, and group 3 late embryogenesis protein) showed significant matches with the common bean lectins (PHAs) (Kasera et al., 2011).

### 2.3 Labeling regulations

People who have food allergies should read labels and avoid foods to which they are allergic. Legislation regulating the packaging and labeling of prepackaged foods containing allergens at the level of food service establishments is essential to ensure food safety. The US government's Food Allergen Labeling and Consumer Protection Act mandates the proper labeling of prepacked foods containing the big eight derived ingredients and these labeling requirements extend to retail and food service establishments that offer products for human consumption (Messina & Venter, 2020). The allergen's food source must be mentioned on the food label at least once in one of two ways, either “flour (wheat),” and “whey (milk)” or “Contains wheat, milk, and soy”. Further, the Food Allergy Safety, Treatment, Education, and Research Act was signed into law on April 23, 2021, including sesame as the 9^th^ significant food allergen recognized by the United States. The new policy has become effective from January 1, 2023 and all United States Food and Drug Administration requirements of labeling and manufacturing a new major food allergen sesame (https://www.fda.gov/food/food‐labeling‐nutrition/food‐allergies). In Europe, 14 major food products are identified as a major source of food allergies. The EU Food Information Regulation and Food Information for Consumers Regulation have several amendments to ensure that allergen labeling laws apply to prepacked and nonprepacked foods (Baker, 2018). Globally, the Codex Alimentarius Commission developed the Codex General Standards for the Labeling of Prepacked foods, listing peanuts, tree nuts, soy, milk, eggs, fish, crustaceans, as well as cereals containing gluten and sulfites. This sets the limit of >10 mg/kg above which products should contain a precautionary labeling statement. The Codex standards guide helps legislative bodies in developing the regulatory frameworks of Codex member countries (FAO, WHO, n.d.). Several national and international organizations, such as Food Allergy Research and Education, aim to enhance food allergen labeling regulations around the world (https://www.foodallergy.org/). The food safety regulatory body of India, the Food Safety and Standard Authority of India, published regulations that comply with Codex Standards specifying packaging and labeling principles for packaged foods containing allergenic constituents (“Food Safety & Standards [Labelling & Display]”, 2020). The food service establishments should provide a declaration on the package regarding food allergens specifying the name of allergy causing ingredients. In the case of packaged foods with cross contaminated ingredients that are known to cause allergies declared “may contain particular allergy causing ingredients.” Whereas, allergen labeling requirements, that is, the declaration is not required in the case of oils and alcoholic beverages derived from these ingredients and raw agricultural commodities (https://www.fssai.gov.in/upload/uploadfiles/files/Compendium_Labelling_Display_23_09_2021.pdf). Further, evaluation of global food allergen labeling laws pertaining to foods and allergens labeling were comprehensively reviewed at the level of county and region, and also emphasize implementation of other protective measures by Chang et al. (2023).

### 2.4 Impact of global trade

Due to the globalization of the market, peanut‐based food product industries have huge export potential. There are no general estimates of direct economic losses in the global food market due to the allergenicity of food commodities. However, the indirect economic costs of FA concerns in global trade can be calculated from the rise in the FA and food intolerance product market. Increasing healthcare awareness has led to a growing preference for allergen‐free, gluten‐free, and lactose‐free diets, which have accelerated the growth of the global FA product market. According to Strategy R (https://www.strategyr.com/market‐report‐food‐allergy‐and‐intolerance‐products‐forecasts‐global‐industry‐analysts‐inc.asp), which is a trademark for global industry analysis, the global market of FA and intolerant products will reach US$32 billion by 2027. It was estimated to be US$22.4 billion in 2020, with a projected compound annual growth rate (CAGR) of 5.2% between 2020 and 2027. The lactose‐free food product segment alone is projected to have a CAGR of 4.6%, reaching US$15.1 billion by the end of 2027. The FA and intolerance products market of the United States was estimated to be US$6.6 billion in 2020, followed. The forecast for China was US$5.7 billion in 2027 at a CAGR of 4.9%. The North American market is growing fast due to a rapid increase in the rate of food allergies and sensitivity. Subsequently, the European and Asia Pacific markets have followed a similar trend due to innovations in food processing industries and a rise in consumer awareness about food safety (https://www.coherentmarketinsights.com/ongoing‐insight/food‐allergy‐and‐intolerance‐products‐market‐788).

## 3 THE FUNCTIONAL BIOLOGY OF ALLERGENS

Humans are constantly exposed to thousands of plant food proteins through ingestion. However, only a limited group of proteins can trigger an allergic response in certain individuals. This intrinsic property of allergenicity is attributed to the biochemical and structural makeup complementary to IgE antibodies. Other factors such as biochemical and molecular properties, such as size, solubility, stability to acidic pH and enzymatic hydrolysis, disulfide bond‐stabilized conformational IgE epitopes, oligomerization, post‐translational modifications, interactions of the protein food matrix and ligand binding, also influence allergenicity (Pekar et al., 2018).

### 3.1 The nature and functions of allergens

Most plant food allergens are seed storage and defense‐related proteins (Breiteneder & Radauer, 2004). Seed storage proteins, which account for approximately 50% of the total protein content, act as the seed nutrient store and are required for germination and seedling growth. Of these, legumins, 2S albumin and vicilins contribute 50–70, 20–60, and 20% of the total protein fraction, respectively (Monsalve et al., 2007). However, there is no relationship between the abundance of seed storage proteins and allergic sensitization. Factors such as the chemical composition of allergens, processing methods, routes of exposure, structural stability, interactions with lipid molecules, aggregation, cross‐reactivity, and patient factors determine the potency of allergenicity (Breiteneder & Mills, 2005; Smits et al., 2018). Plant defense proteins such as pathogenesis‐related (PR) proteins, nsLTPs and profilins protect against invasion by pathogenic microorganisms and herbivory by insect pests, as well as preventing the adverse effects of abiotic stresses (Sinha et al., 2014). PR proteins, such as PR‐10, are induced in response to exposure to biotic and abiotic stresses (Sinha et al., 2014). In addition to their defensive role, nonspecific nsLTPs are required for plant growth and development, cuticle formation, suberin biosynthesis, pollen development, seed maturation and germination, fruit ripening, and defense signaling (D'Agostino et al., 2019; Liu et al., 2015). The profilin superfamily proteins are known to be involved in the reorganization of the actin cytoskeleton and signal transduction by regulating intracellular calcium levels (Asturias et al., 2002).

### 3.2 Classification of plant food allergens

In the AllFam database (http://www.meduniwien.ac.at/allfam/), allergens are classified into protein families based on the data available from the WHO/IUIS Allergen Nomenclature Database and Allergen Online and Pfam databases (Radauer et al., 2008). An AllFam search for plant food allergens with ingestion as a route of exposure revealed 233 allergens that are primarily distributed in allergen super families, such as Prolamin (75), Cupin (36), Profilin (26), PR‐10 protein (21), Thaumatin‐like protein (10), Oleosin (8), Defensins (2) and others (Costa et al., 2020).

#### 3.2.1 The prolamin superfamily

A prolamin superfamily is a major group of allergens, such as 2S albumin, nsLTPs, and cereal alpha‐amylase/protease inhibitors. These allergens are primarily found in rice, wheat, peanuts, brazilnut, and fruits with peaches. They are low‐molecular‐weight proteins that are rich in proline and glutamine. They contain α‐helical globular domains with conserved intramolecular disulfide bonds that are resistant to thermal processing and proteolytic digestion (Breiteneder & Radauer, 2004; Mills et al., 2004). The prolamin superfamily includes cereal prolamin (soluble gliadins and insoluble glutenins), 2S albumin, nsLTPs, bifunctional alpha‐amylase/proteinase inhibitors, soybean hydrophobic proteins related to nsLTP, and indolines, which are cereal antimicrobial proteins that contribute to grain softness. 2S albumin is a predominant seed storage protein. It is widely distributed in the seeds and nuts of dicotyledonous plants. Examples of 2S albumins include Ara h 2, Ara h 6, Ara h 7 from peanut, Gly m 8 from soybean, and the Jug r 1, Cor a 14, Car i 1, Ana o 3, Pis v 1, and Ber e 1 proteins from different tree nuts (Table 1 and Figure 5). In the case of peanut allergens, Ara h 1, 2, 3, and 6 show predominant expression in seeds.

nsLTPs are small (6.5–10.5 kDa), basic proteins that are widely distributed in higher plants and serve many important physiological processes, including defense against bacteria and fungi (D'Agostino et al., 2019). While nsLTPs contain an eight‐cysteine motif backbone, the type 1 and type 2 nsLTPs differ in size and disulfide bonding patterns (Liu et al., 2015; Salminen et al., 2016). nsLTPs bind phospholipids, fatty acids and a variety of hydrophobic molecules. They are commonly found in nuts, celery tubers, seeds, fruits, vegetables, pollen, and latex.

Bifunctional α‐amylase/protease inhibitors are proteinaceous enzyme inhibitors that are primarily found in storage organs such as seeds and tubers. They function in protection against phytophagous insect pests by inhibiting insect gut enzyme activity, thereby hindering the digestion of plant food starch and protein. These proteins possess a small, compact structure rich in disulfide bonds and act as a storage reserve during seed germination. They include Ara h 1, Ara h 2 from peanut, Tri a 28, Tri a 29, Tri a 30, Tri a 33, Tri a 39, and Tri a 40 from wheat (Table 1; Maleki et al., 2003; Figure 5).

#### 3.2.2 The cupin superfamily

The allergenic cupins superfamily are globulin‐type seed storage proteins (Radauer & Breiteneder, 2007). They have a β‐barrel core domain structure and exist as single domain‐cupins and bicupins. They elicit life‐threatening allergic reactions in individuals sensitive to peanuts, soybean, almond, hazelnut, and walnut and are grouped into the legumin and vicilin families (Costa et al., 2020).

Legumins (11S globulin) are abundant (50–70%) seed storage proteins (Mills et al., 2002). They have hexameric structures (360 kDa each) linked by noncovalent interactions. They are composed of six monomeric units derived from the respective gene products. Members of 11S globulin allergens include peanut Ara h 3, soybean Gly m 6, walnut Jug r 4, almond Pru du 6, hazelnut Cor a 9, pecan Car i 4, cashew Ana o 2, pistachio Pis v 2, Pis v 5, and brazil nut Ber e 2 (Table 1 and Figure 5).

Vicilins (7S globulin) are abundant seed storage proteins (up to 20%) that are often found in legumes and tree nuts. They exist as trimeric proteins (150–190 kDa) that can aggregate into hexamers. They differ from legumins because they lack disulfide bonds. Examples include the peanut Ara h 1, soybean Gly m 5, walnut Jug r 2, Jug r 6, hazelnut Cor an 11, pecan Car i 2, cashew Ana o 1, and pistachio Pis v 3 (Table 1; Geiselhart et al., 2018).

#### 3.2.3 Profilin superfamily

Profilins are highly conserved, cross‐reactive cytosolic panallergens (12–15 kDa) that are primarily found in pollen and latex. Sensitization occurs because of cross‐reaction with IgE antibodies (Asero et al., 2003). Major allergens of the profiling family were reported in the peanut Ara h 5, soybean Gly m 3, wheat Tri a 12, walnut Jug r 7, almond Pru du 4, and hazelnut Cor a 2 (Table 1 and Figure 5).

#### 3.2.4 Pathogenesis‐related (PR‐10) proteins

PR‐10 proteins (15–17 kDa) are mostly found in fruits and vegetables but they are also the cause of birch pollen‐associated allergies (Sinha et al., 2014). They are structurally distinct from other PR proteins because they contain highly conserved seven‐stranded antiparallel β‐sheets surrounding α‐helix at the C‐terminus (Fernandes et al., 2013). Many birch pollen allergic individuals are also sensitive to multiple fruits and vegetable allergens because of Bet v 1 cross‐reactive IgE antibodies. Symptoms ranging from mild to potentially life‐threatening conditions have been reported following the consumption of raw foods (Costa et al., 2020).

## 4 APPROACHES TO REDUCE ALLERGEN CONTENT

Most plant food allergens are water‐soluble glycoproteins that are relatively stable with regard to enzymatic hydrolysis, heat, and chemical treatments (Sicherer & Sampson, 2010). However, different food processing methods can modify, at least in part, immunogenic reactivity (Bhalla & Singh, 2008). Such methods have both limitations and advantages in reducing the allergenic effect but may decrease the nutritional value of food (Fu et al., 2019). Most studies used to evaluate processing methods depend on in vitro IgE binding assays rather than more relevant *ex vivo* basophile activation tests (BAT) and mast cell activation tests (Shah et al., 2019).

### 4.1 The genomics of allergens

In the last decade, more than 35 different species of legumes have been sequenced for developing reference genome and transcriptome assemblies. This genomic resource information accelerates the development of cultivars of superior grain legume crops by genomic‐assisted breeding and precision breeding (Bauchet et al., 2019; Varshney et al., 2019). Similarly, genetic approaches are being used increasingly to develop low allergen food crops (Zhou et al., 2013). For example, a lower Gly m Bd 30K (P34) allergen was identified in soybean (Jeong et al., 2013). The high‐quality RefSeq (reference sequence) v1.0 reference genome from the International Wheat Genome Sequencing Consortium was used to detect and properly discriminate allergens and antigens in wheat proteins linked to or involved in human disease (Juhász et al., 2018). While relatively few quantitative trait locus or association studies have been conducted in other species, the expression levels of allergens can be altered through molecular breeding and/or genetic engineering using RNA interference (RNAi) or clustered regularly interspaced short palindromic repeats (CRISPR)/Cas9 mediated gene editing (Jouanin et al., 2018; Saurabh et al., 2014). A hypoallergenic peanut variety that was produced by gene silencing showed a 25% reduction in the expression of the most potent allergen Ara h 2, thereby significantly decreasing allergenicity (Dodo et al., 2008). Studies involving the silencing of the Ara h 1, Ara h 2, Ara h 3, and Ara h 6 genes resulted in a significant reduction in IgE binding. Crucially, no changes in seed weight or germination were observed between the transgenic and wildtype plants (Chu et al., 2008). Several peanut lines with lower levels of the major allergens (Ara h 1, Ara h 2, Ara h 3, Ara h 6, and Ara h 8) were identified at ICRISAT using a large‐scale phenotyping approach (Pandey et al., 2019). Such studies can lead to the identification of functional variations that may be useful in molecular breeding approaches involving marker‐assisted selection and marker‐assisted backcrossing (Janila et al., 2016; Varshney et al., 2014). Similarly, RNAi approaches have been successfully used to mitigate the expression of the allergen *Gly m Bd 30k* protein in soybean (Herman, 2003) and rice (Ogo et al., 2014) and apple (Dubois et al., 2015). In addition, CRISPR/Cas9‐mediated site‐directed mutagenesis was successfully used to eliminate two major allergen genes, Gly m Bd 28k and Gly m Bd 30k and thus generate hypoallergenic soybeans (Sugano et al., 2020). The gene silencing studies of Barro et al. (2016) selectively targeted the gliadin and glutenin genes using RNAi technology. An absence of epitopes related to coeliac disease related to immunogenic gliadins was reported in the wheat lines. Similarly, a low‐gluten wheat variety that showed an 85% decrease in immunoreactivity was produced (Sánchez‐León et al., 2018). Such lines could be used as source material for further introgression studies. Efforts have been made in durum wheat to edit the genes encoding α‐amylase/trypsin inhibitors (ATIs) that are involved in wheat allergy and nonceliac wheat sensitivity (Camerlengo et al., 2020). Such studies illustrate the enormous potential of molecular genetic approaches to produce hypoallergenic food crops. However, the removal of allergenic seed storage proteins on a large scale may result in large decreases in nutritive value and taste.

### 4.2 Food processing methods

#### 4.2.1 Physical processing methods

Thermal processing, such as frying, roasting, curing, and various types of cooking, can result in a variety of nonenzymatic, biochemical events in meals. Many foods brown due to a phenomenon known as the Maillard reaction, which is one of the key processes that occur during food cooking or browning (e.g., roasting, frying, and curing). The Maillard reaction is an important in the development of flavour and colour in foods such as peanuts and tree nuts during roasting, enhances flavours in beverages such as beer and coffee, and involves a process similar to caramelization in which amino groups of proteins are modified via nonenzymatic condensation with reducing sugars. Each method has a varied effect on the allergenic potency of different foods. Processing approaches involve include the use of physical methods (heat, mechanical, electric, and magnetic energy) that disrupt protein structure and induce aggregation but without disrupting the primary structure. The most common physical methods are thermal processing, irradiation, ultrasound, ultrahigh pressure, and microwaves (Cabanillas et al., 2018; Vanga et al., 2017; Verhoeckx et al., 2015). Such methods have been widely applied to reduce allergenicity. They have advantages over chemical and enzymatic methods in terms of cost, time, side effects, and nutritional quality. Boiling, roasting, frying, and autoclaving are the most common methods of household food preparation. While boiling can decrease the immunoreactivity of allergenic protein, roasting increases allergenicity (Kopper et al., 2005; Turner et al., 2014). In the case of peanuts, for example, roasting enhances IgE binding activity by 90‐fold and makes the allergens Ara h 1 and Ara h 2 resistant to digestive enzyme proteolysis because of Maillard reactions (Maleki et al., 2000). Thermal processing can therefore reduce immunogenicity to a certain degree but it may also destroy nutrients and bioactive ingredients (Gupta et al., 2018b).

Structural denaturation, unfolding, glycation, and aggregation occur during the physical processing of food proteins. This affects solubility and digestibility, which might lead to the elimination of conformational IgE epitopes or the formation of new allergenic linear epitopes that can increase the risk of allergy (Shah et al., 2019; Verhoeckx et al., 2015). The application of ultrasound to reduce the allergenicity of certain food products has proved to be a useful pretreatment before food processing (Corzo‐Martínez et al., 2017). In addition, the application of a pulsed ultraviolet light was able to decrease the levels of glycinin and β‐conglycinin allergens in soybean (Yang et al., 2010). Unfortunately, there are no inconsistent food processing methods that reduce allergenicity in different food materials. Moreover, the conventional thermal processing methods applied in the reduction of food immunoreactivity significantly destroy nutritional components present in food sources. Therefore, the use of novel nonthermal processing techniques including high‐pressure processing, ultrasound, pulsed light, cold plasma, fermentation, pulsed electric field, and enzymatic hydrolysis generally have better performance in retaining the original characteristics of food and improving the efficiency of eliminating allergens (Dong et al., 2021).

#### 4.2.2 Chemical and enzymatic methods

The physical methods discussed for food processing affect the physicochemical properties of food proteins in diverse ways and influences their gastrointestinal digestion, bioavailability, and allergenicity. However, the application of nonthermal including chemical and enzymatic methods can induce minimal changes to food quality attributes and can extend the shelf‐life of food (Dong et al., 2021; Ekezie et al., 2018). Chemical and enzyme treatments can reduce or destroy immunogenic determinants in food by disrupting the allergen structure that is stabilized by covalent and noncovalent bonds (Ekezie et al., 2018; Wang et al., 2022). Acid hydrolysis is widely used to treat wheat flour and destroy gluten allergen proteins to produce low allergenic products (Fu et al., 2019). Major covalent modifications, such as acylation, reduction, and alkylation, show remarkably reduced immunogenicity by altering the solubility and digestibility of allergens in the gastrointestinal tract (Apostolovic et al., 2013). In addition, non‐covalent modifications that involve binding with compounds such as phytic acid, phenolic compounds, and tannic acid to form insoluble complexes have been shown to decrease allergic potency in peanuts by hindering proteolytic digestion (Chung & Champagne, 2008; Chung & Reed, 2012). Furthermore, polyphenol‐enriched peanut matrices were shown to significantly minimize allergen interactions with IgE and decrease *ex vivo* basophil degranulation and mast cell degranulation in a mouse model (Plundrich et al., 2017). This phenomenon involved excessive proteolytic digestion of the polyphenol–allergen complex, which facilitated alterations in conformational epitopes and the simultaneous masking of linear epitopes. However, the addition of phenolic compounds and polyphenols can cause stomach discomfort and it also obstructs nutrient absorption in the intestine.

Enzymatic hydrolysis has shown promising results with regard to reducing allergenicity. Cross‐linking of enzymatic proteins with allergens masks antibody‐specific epitopes. In contrast, proteolysis with food‐grade enzymes, such as trypsin, chymotrypsin, papain, ficin, bromelain, and so on, disrupts the native structure and physiochemical characteristics, as well as IgE‐specific conformational and linear epitopes, which ultimately reduces allergenicity (Meng et al., 2020; Zhou et al., 2013). The roasted peanut allergens Ara h 1 and Ara h 2 are completely hydrolyzed by treatment with trypsin (0.15%) and chymotrypsin (0.1%) for 3 h (Yu et al., 2011). Similarly, Ara h 1, Ara h 2, and Ara h 3 are effectively eliminated by hydrolysis using alcalase and flavorzyme (Cabanillas et al., 2012).

Enzymatic hydrolysis followed by physical processing methods, such as irradiation, pulsed ultraviolet light, pulsed electric field, high‐pressure processing, and high‐intensity ultrasound have been proven to be effective in reducing allergenicity (Shah et al., 2019). However, the combination of autoclaving and fermentation of raw peanut pulp with *Bacillus natto* effectively diminishes the allergens in raw peanuts (Pi et al., 2021). Such effects have also been demonstrated in wheat for gluten‐free bread (Diowksz & Leszczyńska, 2014).

The combination of physical methods and enzymatic hydrolysis (hurdle technologies) has the advantage of facilitating efficient enzyme penetration and proteolysis. Similarly, the chemical reduction of disulfide bonds in allergenic proteins destabilizes the three‐dimensional structure and increases the efficiency of enzymatic proteolysis (Mikiashvili & Yu, 2018). Unfortunately, however, the majority of food processing methods also alter the texture and flavor of food and this can significantly affect consumer acceptance.

### 4.3 Common methods for allergy diagnosis

There are various diagnostic methods available for testing allergies which involve skin or blood. Allergy testing assesses the body's reaction to specific allergens and the test must be chosen by a trained health professional called an allergist based on symptoms, age, hobbies, exposures and patient medical history. Such testing reveals allergens that might cause allergies, such as plant pollens, molds, dust mites, animal dander, insect stings, and various foods such as peanuts, eggs, wheat, shellfish, and milk and also includes some medicines like penicillin. Once the allergens have been identified through proper diagnostic methods, the specific treatments can be possible through medications, allergen immunotherapy, and/or environmental control measures to achieve long‐term sustainable outcomes (Ansotegui et al., 2020; Dreborg, 2001; Heinzerling et al., 2013; Maruyama, 2021).

#### 4.3.1 Skin prick/scratch test (SPT)

It uses a thin needle to prick the skin on your forearm or back with a possible number of different potential allergens or the allergist may place droplets of potential allergens onto your skin and use a device to scratch and lightly puncture the area. It helps the liquid to enter into the skin and is observed for the body's reactions might be a rash or round spots which are generally used for the detection of airborne allergies, food allergies and penicillin allergies. The skin prick test represents the most reliable and cost‐effective tool for the diagnosis and management of IgE‐mediated allergy.

#### 4.3.2 Intradermal skin test

If skin prick test results turn inconclusive, a small amount of the allergen is injected into the epidermis and records the observations. This test is used for the diagnosis of allergies to airborne irritants, insect stings and medications.

#### 4.3.3 Patch test

The purpose of this test is to determine the cause of contact dermatitis in which a patch of allergen‐containing bandage is applied on to the skin. After 2–3 days the allergist records the observation of allergic reactions.

#### 4.3.4 Blood (IgE) test

When skin tests are inconvenient for a particular patient, an allergist can proceed with the blood test. This test measures levels of allergen‐responsive antibody IgE in the serum by the addition of different potential allergens to the blood.

#### 4.3.5 Basophile activation tests

This laboratory test measures the activation of basophils, a type of red blood cells in response to a specific allergen. Further, the BAT is specific which allows better defining the IgE profile of the patient but it is complex to perform.

#### 4.3.6 Challenge tests

This test is particularly used to identify the source of food allergies. Under the supervision of a health professional, the person with suspected FA ingests a small amount of an allergen and the allergist observes the symptoms of allergic reaction. This test is highly risky for the individual sensitive to anaphylaxis which required immediate epinephrine injection to stop the reaction.

Among all the allergy testing methods, the skin test is the gold standard and is used along with a person's medical history to identify the source of FA. While blood tests generally have a higher rate of false‐positive results, in addition to the pain and chances of bleeding.

## 5 SCOPE FOR DEVELOPING HYPOALLERGENIC CROPS WITH MINIMAL EFFECTS ON PLANT PHYSIOLOGY

Most allergens are seed storage proteins that play a key role in plant biology, with functions ranging from seed germination to defense against biotic and abiotic stresses (Zhou et al., 2013). Therefore, the selection of target genes of allergens in particular crop plants requires a comprehensive understanding of gene function in plant growth and development.

Of the 18 recognized allergens in peanuts, Ara h 1, Ara h 2, Ara h 3, and Ara h 6 (Shah et al., 2019; Wu et al., 2016), Ara h 2 is recognized in most peanut‐allergic individuals. Therefore, a reduction in peanut allergy by eliminating the Ara h 2 genes might be a preferable choice that might not result in major alterations to plant growth and development. Efforts have been made to silence Ara h 2 and Ara h 6 in peanuts using RNAi technology. Such approaches have resulted in a significant decrease in IgE binding with no significant effects on seed germination or defenses against fungal infection (Chu et al., 2008). Other studies have also sought to decrease peanut allergy by silencing Ara h 2 using a specific RNAi gene silencing (Dodo et al., 2008). Transgenic peanut lines with suppressed Ara h 2 and Ara h 6 protein expression remained stable for several generations (Chandran et al., 2015). In the case of wheat, silencing of gluten synthesis led to the production of a low gluten wheat variety, which is safe for many gluten allergy‐sensitive individuals (Wen et al., 2012). Similarly, the ω−5 gliadin‐free wheat line 1BS‐18 had low efficiency in inducing allergy symptoms in guinea pigs (Kohno et al., 2016).

Soybeans contain two major allergens in the form of 7S globulin (β‐conglycinin) and 11S globulin (glycinin). These together make up >50% of the total seed protein. Suppression of globulin and conglycinin expression using RNAi did not affect seed size, weight or developmental ontogeny. However, these soybean lines undergo were found to express other seed proteins (Schmidt et al., 2015). Another study using microRNAs specific to 7S globulin had no adverse effects on seed lipid, carbon and nitrogen contents (Yamada et al., 2014). CRISPR/Cas9 gene editing was used to create a double mutant (Gly m Bd 28 K and Gly m Bd 30 K) which resulted in the loss of both proteins from Japanese soybean seeds (Sugano et al., 2020). Taken together, such studies provide substantial evidence that plants show a compensatory response to the suppression or elimination of allergenic seed proteins. The success of such studies demonstrates the potential of such current gene technologies for the creation of hypoallergenic plant foods.

## 6 PHENOMICS AND OMICS APPROACHES TO REDUCING ALLERGENS

### 6.1 Identification of crops with reduced allergen content through selection and breeding

Conventional plant breeding plays a significant role in developing new plant varieties with desired plant traits/features. Unfortunately, due to lack of information on low allergen content lines, there is not much effort on breeding varieties with reduced allergen content using conventional breeding approaches (Pandey et al., 2019; Riascos et al., 2010). Limited conventional breeding efforts are reported to improve the nutritional content of crops, which indirectly contributes to reducing allergenicity (Lemke et al., 2022). By increasing the nutritional value of crops, individuals with food allergies may have access to a wider range of nutrients and alternative food sources (Kaiser et al., 2020). Plant food allergens are not always a strict selection criterion comparable to other plant toxins, especially considering that food allergens are always unique proteins of large protein families with complex inheritance in plant breeding. Although screening germplasm to identify individuals with decreased allergen content is time‐consuming, traditional breeding attempts toward hypoallergenic variants in peanut (Pandey et al., 2019; Perkins et al., 2006), wheat (Yamada et al., 2022), and soybean (Gao et al., 2012) have been attempted. Gluten in hexaploid bread wheat is made up of numerous distinct proteins, the most prominent of which are glutenin and gliadin. Glutenins are essential for baking quality, but gliadins include the majority of celiac disease‐associated pieces (epitopes). Although old hexaploid bread and tetraploid durum wheat varieties have been identified with few epitopes connected to gluten intolerance, generating favourable combinations of gluten genes to satisfy baking quality standards in a polyploid is difficult (Gilissen et al., 2014; Lemke et al., 2022).

Little information is available concerning the major allergen contents of peanut germplasm lines that are commercially grown around the world. Recently, Pandey et al. (2019) identified hypo‐allergenic lines for Ara h 1, Ara h 2, Ara h 3, Ara h 6, and Ara h 8. These and other studies have used monoclonal antibodies to screen peanut‐based products (Filep et al., 2018). Earlier, 34 peanut genotypes were screened using patient sera but no substantial differences in allergen content were identified (Dodo et al., 2002). The analysis of a “Reference set” consisting of 300 genotypes representing 48 countries has also been reported (Upadhyaya et al., 2003, 2010).  Little variation was observed in 53 Chinese peanut cultivars (Wu et al., 2016) using human sera to assess the allergen content in their cultivars. However, the Spanish bunch varieties had lower peanut allergen contents than the other agronomic types. These authors also reported that Xinxiandahuasheng of the Virginia type, Bangjihonghuasheng of the Valencia type, Mangdou of the Spanish type, and Yaoshangxiao make of the Peruvian type had lower peanut allergen contents. A study of 35 US peanut cultivars using antisera from allergic patients also found no significant variation (Dodo et al., 2002; Isleib & Wynne., 1992). However, rapid and easy phenotyping methods for different allergens are required to increase the efficiency of breeding reduced allergen crops (Liu et al., 2023).

The pattern of sensitization to peanut allergens varies in different geographical regions (Vereda et al., 2011).  For example, Ara h 1, Ara h 2, and Ara h 3 were identified in allergic reactions in the United States. Similarly, Ara h 1, Ara h 2, and Ara h 3 11 were found in European nations (Ballmer‐Weber & Beyer, 2018). Nine soybean allergens were identified in three soybean varieties developed at nine locations in three states in the same climate zone in North America: Illinois, Iowa and the United States (Mcclain et al., 2018).

Mutation breeding has also been shown to be effective in a wide range of crop species, such as tomato, rapeseed, cotton, barley, sunflower, peanut, cassava, and can be successfully used to improve plant varieties (Xia et al., 2022). Mutation breeding has been used to increase the yield and oil and protein contents of peanuts (Hamid et al., 2006). A Targeting Induced Local Lesions IN Genomes approach was used to create gene‐specific primers for each of the two Ara h 1 and two Ara h 2 genes to find mutations in peanuts (Knoll et al., 2011). Similarly, gamma irradiation mutagenesis was used to identify the mutant alleles of the Gly m Bd 28 K and Gly m Bd 30 K genes in soybean.

### 6.2 Role of artificial intelligence and bioinformatics in reducing allergencity

Traditional methods for identifying food allergens mostly rely on in vivo and in vitro experiments, which can be time consuming and uneconomical. However, artificial intelligence (AI) and bioinformatics have the potential to significantly reduce plant food allergens by aiding in the identification of specific allergenic proteins in plants as well as the development of novel methods to modify or remove allergens from food products (Liu et al., 2023). AI in allergy and immunology has various potential therapeutic applications ranging from disease diagnosis to multidimensional data reduction in electronic health records or immunologic datasets (Khoury et al., 2022). The applications of AI and bioinformatics can be deployed in the prediction of allergenicity by analysing available genomic and proteomic data. By analyzing the genetic information of various plant species, AI algorithms can identify potential allergenic proteins and predict their structure and function. One of the study proves quick food allergen identification approach powered by AI is now a useful auxiliary tool for the prediction of allergenicity of food proteins using deep learning models by overcoming the limitations of low accuracy traditional machine learning models (Wang et al., 2021). A novel chemometric method for analysing and investigating the allergenic properties of dietary proteins has been developed by using machine learning. The approach is based on rating descriptors and evaluating their classification performance. It is necessary to create a reliable and effective protein categorization system in order to overcome the issue of food allergies (Nedyalkova et al., 2023). This information can then be used to guide the selection of plant varieties and to design breeding strategies that reduce the expression of allergenic proteins or to develop targeted approaches for modifying allergenic proteins. Further, bioinformatics can potentially analyze large datasets of allergen information and identify common allergenic epitopes across different plant species. This can help researchers identify potential targets for developing plant varieties with reduced allergen content. This information can be used to design novel food processing techniques that can reduce the allergenicity of plant foods. For instance, AI algorithms can be used to model the effects of different processing conditions on the structure and function of allergenic proteins, allowing researchers to identify conditions that reduce allergenicity without compromising the nutritional quality or taste of the food. Further, AI can help researchers optimize the production process of hypoallergenic plant products by analyzing factors such as temperature, humidity, and nutrient levels. By analyzing the structure and function of allergenic proteins, AI algorithms can identify potential targets for genetic engineering or protein modification that can reduce allergenicity while maintaining the nutritional value of the food. The scope of AI and bioinformatics for reducing plant food allergens is vast and includes applications in genetic analysis, allergen identification, and production optimization (MacMath et al., 2023; Solanki et al., 2020). These technologies have the potential to revolutionize the field of plant food allergen reduction, leading to safer and more accessible food for individuals with allergies for their health and well‐being.

### 6.3 Developing varieties with reduced allergen content using genetic engineering and gene editing technologies

The genetic engineering approach RNAi is a powerful tool that can be used to improve various traits in crops, including nutrient value, disease or pathogen resistance, and crop allergenicity reduction. RNAi is a natural biological process that regulates gene expression by suppressing the activity of specific genes. Further, integrating RNAi technology with conventional breeding approaches contributes to the development of improved crop varieties that address nutritional, health, and environmental challenges (Rajam, 2020). By using RNAi, the expression of allergen proteins can be reduced or eliminated, resulting in crops with reduced allergenic potential and this approach holds promise for improving the safety of food products for individuals with food allergies. A significant progress has been made in gene silencing approaches to suppress the immunodominant allergen Ara h 2 in peanuts (Dodo et al., 2008). Decreased allergenicity to the immune dominant Ara h 2 protein was achieved in peanuts using RNAi technology (Dodo et al., 2008). In addition, RNAi approaches have been successfully used to suppress the expression of Ara h 2 and Ara h 6 allergens without adverse effects on seed germination and plant growth and development (Chandran et al., 2015; Chu et al., 2008). Similarly, the silencing of gluten synthesis in wheat led to the production of a low‐gluten wheat variety (Wen et al., 2012). Moreover, the ω−5 gliadin‐free 1BS‐18 wheat line had low efficiency in inducing allergy symptoms in guinea pigs (Kohno et al., 2016).

In addition to RNAi, the use of technological advancement, the discovery and characterization of plant food allergen genes offers a significant opportunity for successful genetic modifications (Brackett et al., 2022). Biotechnological techniques, such as gene editing or genome editing, are widely employed to produce designer crops with desired traits (Wang et al., 2016; Fernie and Yan, 2019; Awasthi et al., 2022). Site‐directed nuclease (SDN) methods have also recently been employed to achieve genetic alterations by a precise cleavage in the intended target region of the genome. A few SDN tools, such as zinc‐finger nucleases (ZFN) (Urnov et al., 2010), transcription activator‐like effector nucleases (TALEN) (Joung & Sander, 2013), and CRISPR (Wang et al., 2016) are currently used for gene editing (Camerlengo et al., 2020; Kaur et al., 2020; Lakhani et al., 2022; Singh et al., 2023).

CRISPR/Cas9 is a well‐proven technology useful for creating desirable mutations at specific genetic locations that can also be applied for key polyploid crops, such as peanut and wheat, and diploid crops, such as soybean, kidney beans, and mustard (Assou et al., 2022; Bortesi & Fischer, 2015; Gao, 2021; Steinwand & Ronald, 2020; Weeks, 2017). CRISPR/Cas9 has been used successfully to edit the Ara h 2 genes in peanuts (Biswas et al., 2022). This technology has also been successfully used to edit fatty acid desaturase (*AhFAD2*) in peanuts to increase the oleic content (Yuan et al., 2019). Similarly, the roles of nod factor receptors (*AhNFRs*) were verified by using CRISPR/Cas9 (Shu et al., 2020). The research group of Rustgi et al. (2022) has developed low‐gluten producing wheat by expressing glutenases through genetic engineering. They have also screened selected genotypes of the USDA and ICRISAT mini‐core of peanuts for low allergenic content and targeted the major allergen genes by CRISPR/Cas approaches.

The successful genome editing of *GmDcl4a* and *GmDcl4b* genes was reported in hairy roots (Curtin et al., 2011). Recently, two major soybean allergens, Gly m Bd 28 k and Gly m Bd 30 k, were removed from seeds using CRISPR/Cas9‐mediated site‐directed mutagenesis (Sugano et al., 2020). The CRISPR/Cas9 system was also used to knock out the *TaMLO* locus in wheat (Shan et al., 2013), including *TaPDS* and *TaINOX* (Upadhyay et al., 2013). The three *TaMLO* alleles were silenced together for resistance to powdery mildew in bread wheat (Wang et al., 2014). The wheat *TaLOX2* gene was silenced by expressing sgRNA under the transcriptional control of the TaU6 promoter (Shan et al., 2013). The major allergenic proteins ATIs were recently silenced using the CRISPR/Cas9 approach, in order to decrease wheat allergies such as Baker's asthma and nonceliac sensitivity (Camerlengo et al., 2020).

## 7 SUMMARY AND FUTURE PROSPECTS

Legume food crops are the major source of essential amino acids and plant‐based proteins besides their natural antinutrients and allergenic substances that hampers digestibility and trigger an abnormal immune response which makes them undesirable for human consumption. The implementation of novel approaches like high throughput phenotyping, target gene identification assisted by AI, genomics‐assisted selection, and precision breeding by gene editing can help to address the challenges of food safety and security. In the post‐genomic era, researchers have made enormous progress in genome sequencing and structural analysis of legume genomes by the intervention of NGS and bioinformatics. These genome information resources are facilitating gene discovery and development of molecular markers of complex traits toward the generation of superior legume crops. Particularly, in the matter of food safety, allergies have become a serious health concern in both developed and developing countries in the era of modernization. The increasing prevalence of FA presents a formidable challenge to researchers seeking to find accurate, rapid diagnostic methods, as well as prevention and treatment measures for vulnerable people.

Although, a variety of thermal and nonthermal food processing methods have been applied by the food service establishments, however, removal of allergenic substances from the food source material is still a challenge for packaged foods. Therefore, the combined use of technological advancement in AI, bioinformatics, molecular breeding, RNAi, and CRISPR/Cas‐based gene editing can help to develop varieties of legume crops with reduced allergen. Realizing the strength of currently available tools and technologies, developing allergen‐free crop varieties seems very difficult; nevertheless, the available genetic variation among diverse germplasm can be exploited to accumulate superior alleles promoting production of low allergen content in seeds for ensuring food safety. On the other hand, the best possible cure needs to be explored to treat affected patients in addition to developing tolerance from childhood through mini‐exposure to diverse food including causing allergenicity.

## AUTHOR CONTRIBUTION

**Vadthya Lokya**: Data curation; investigation; methodology; writing—original draft. **Sejal Parmar**: Data curation; investigation; methodology; writing—original draft. **Arun K. Pandey**: Data curation; writing—original draft. **Hari K. Sudini**: Investigation; methodology; writing—review and editing. **Dongxin Huai**: Investigation; methodology; writing—review and editing. **Peggy Ozias‐Akins**: Data curation; investigation; methodology; writing—review and editing. **Christine H. Foyer**: Investigation; methodology; writing—review and editing; **Chogozie Victor Nwosu**: investigation; methodology; writing—review and editing. **Barbara Karpinska**: Investigation; methodology; writing—review and editing. **Alison Baker**: Investigation; methodology; writing—review and editing. **Pei Xu**: Data curation; methodology; writing—review and editing. **Boshou Liao**: Data curation; methodology; writing—review and editing. **Reyazul Rouf Mir**: Data curation; investigation; methodology; writing—review and editing. **Xiaoping Chen**: Data curation; methodology; writing—review and editing. **Baozhu Guo**: Data curation; methodology; writing—review and editing. **Henry T. Nguyen**: Data curation; investigation; methodology; writing—review and editing. **Rakesh Kumar**: Data curation; methodology; writing—review and editing. **Sandeep K. Bera**: Data curation; investigation; methodology; writing—review and editing. **Prashant Singam**: Data curation; methodology; writing—review and editing. **Anirudh Kumar**: Data curation; methodology; writing—review and editing. **Rajeev K. Varshney**: Investigation; methodology; writing—review and editing. **Manish K. Pandey**: Conceptualization; data curation; funding acquisition; investigation; project administration; resources; supervision; writing—review and editing.

## FUNDING

The authors are thankful to the Department of Biotechnology, India; National Agricultural Science Fund (NASF) of the Indian Council of Agricultural Research (ICAR), India; Bill & Melinda Gates Foundation (BMGF), USA and MARS‐Wrigley, USA for partial financial assistance to this study.

## CONFLICT OF INTEREST STATEMENT

The authors declare there is no conflict of interest.

## Data Availability

Not applicable.
